# Are we asking the right questions? Working with the LGBTQ+ community to prioritise healthcare research themes

**DOI:** 10.1186/s40900-021-00298-7

**Published:** 2021-09-24

**Authors:** Sally Crowe, Eleanor Barker, Meg Roberts, Lucy Lloyd, Clara M. de Barros, Ben Rebelo-Harris, Catherine Meads, Catherine L. Saunders

**Affiliations:** 1 Crowe Associates Ltd, Oxford, UK; 2 Medical Library, University of Cambridge, Cambridge, UK; 3 University of Cambridge, Cambridge, UK; 4 Primary Care Unit, Department of Public Health and Primary Care, University of Cambridge, East Forvie Building, Forvie Site, Robinson Way, Cambridge, CB2 0SR UK; 5 Research Partners, Research Partners, UK; 6 Faculty of Health, Education, Medicine and Social Care, Anglia Ruskin University, Cambridge, UK

**Keywords:** Research priority setting, LGBT (Q+), Public involvement, Research priorities, Rapid review

## Abstract

**Background:**

Conversations about research priorities with members of the public are rarely designed specifically to include people who identify as Lesbian, Gay, Bisexual, Transgender and Queer (LGBTQ+) and are not researchers.

**Methods:**

Generally, to address this gap, and specifically, to inform future research for CLS, we carried out a rapid review of published research priority sets covering LGBTQ+ topics, and an online workshop to prioritise identified themes.

**Results:**

*Rapid review: results.*

The rapid review identified 18 LGBTQ+ research priority sets. Some focussed on specific populations such as women or men, younger or older people or people living within families. Five addressed transgender and gender non- conforming populations. All of the research priority sets originated from English-speaking, high and middle-income countries (UK, US, Canada, and Australia), and date from 2016 onwards. Prioritization approaches were wide-ranging from personal commentary to expert workshops and surveys. Participants involved in setting priorities mostly included research academics, health practitioners and advocacy organisations, two studies involved LGBTQ+ public in their process. Research priorities identified in this review were then grouped into themes which were prioritised during the workshop.

*Workshop: results.*

For the workshop, participants were recruited using local (Cambridge, UK) LGBTQ+ networks and a national advert to a public involvement in research matching website to take part in an online discussion workshop. Those that took part were offered payment for their time in preparing for the workshop and taking part. Participants personal priorities and experiences contributed to a consensus development process and a final ranked list of seven research themes and participants’ experiences of healthcare, mental health advocacy, care homes, caring responsibilities, schools and family units added additional context.

**Conclusions:**

From the workshop the three research themes prioritised were: *healthcare services delivery*, *prevention,* and *particular challenges / intersectionality* of multiple challenges for people identifying as LGBTQ+. Research themes interconnected in many ways and this was demonstrated by the comments from workshop participants. This paper offers insights into why these priorities were important from participants’ perspectives and detail about how to run an inclusive and respectful public involvement research exercise. On a practical level these themes will directly inform future research direction for CLS.

**Supplementary Information:**

The online version contains supplementary material available at 10.1186/s40900-021-00298-7.

## Plain English Summary

Conversations about research priorities with members of the public who identify as Lesbian, Gay, Bisexual, Transgender and Queer (LGBTQ+) and are not researchers are not common.

We reviewed published research priorities covering LGBTQ+ topics and held an online workshop. The review identified 18 LGBTQ+ research published priority sets. Some focussed on specific populations such as women or men, younger or older people or people living within families. Five were on transgender and gender non- conforming populations. Priorities were achieved by different methods such as workshops and surveys. People involved in setting priorities mostly included researchers, health practitioners and advocacy organisations, two studies involved LGBTQ+ public in their process. Research priorities identified in the review were grouped into themes which were prioritised during the workshop.

For the online workshop, participants were recruited using local (Cambridge, UK) LGBTQ+ networks and a national advert and offered payment for their time. Participants personal priorities and experiences contributed to agreeing a final list of seven research themes in priority order. Participants’ experiences of healthcare, mental health advocacy, care homes, caring responsibilities, schools and family units were helpful.

From the workshop the three top research themes were: *healthcare services delivery*, *prevention,* and *particular and multiple challenges* of people identifying as lesbian, gay, bisexual, transgender or queer. Research themes interconnected in many ways as shown by the comments from workshop participants. This paper describes why these priorities were important from participants’ perspective and offers information about how to run an inclusive and respectful public involvement research exercise.

**Supplementary Information:**

The online version contains supplementary material available at 10.1186/s40900-021-00298-7.

## Background

The aim of this research was to orientate future research projects and funding applications, (primarily for CLS), and to develop research priorities in collaboration with LGBTQ+ public, mainly through an online workshop informed by a rapid review. This article describes the process and outcomes of the exercise, with reflections from the team and public participants. This work was not intended to produce a generic LGBTQ+ healthcare research priorities list.

Regarding terminology, in this article we refer to Lesbian, Gay, Bisexual, Transgender, Queer plus (LGBTQ+) public participants. The plus including other forms of identification such as non-binary, asexual and intersex. Elsewhere in the article we refer to sexual and gender minority health in terms of research priorities. Public involvement is used as a short hand phrase to describe a process of working with the public as part of a research process, not as participants of a research study.

Research agendas are often set by public policy, health professionals, guideline developers, researchers and research funders. Less common is the clear and inclusive involvement of the public in research priority decisions. This can lead to research priorities that don’t necessarily reflect the needs and preferences of the public [1, 2].

However, the recent growth in reports and descriptions of research priority setting suggests that working in partnership with the public (patients, carers, service users etc.) to determine research priorities is becoming more common, especially with the adoption of frameworks that set out clear methods and considerations for equitable participation [3]. Examples of frameworks include: James Lind Alliance [4]; Dialogue Model [5]; surveys [6], Q Sort [7]; and more recently online ‘crowd sourcing’ approaches [8]. Common features of frameworks for research priority setting with the public are: stated values and expectations; guidance on and considerations for involving the public; a process flow of steps to describe and prioritise research questions or themes and a plan for what happens to priorities [9].

There is evidence of health inequalities in LGBTQ+ populations, for example in mental health [10], suicide [11], substance use [12], and health screening [13]. These health inequalities have activated calls to highlight evidence gaps for future research and policy consideration, but also address the needs and concerns of LGBTQ+ people using healthcare systems. While there is strong evidence describing large disparities in health and healthcare outcomes [14], detailed evidence on how to address these disparities still emerging, not least because of limitations in sexual orientation collection and monitoring in health data used for research. Prioritisation work with public involvement is required to understand where research is most needed.

Published existing research priority sets can help researchers coming to an area of interest assess what appears to be important and to whom. Assembling these publications in a review (scoping or systematic reviews) [15, 16] provides further clarity. Combining a review of research priority sets in LGBTQ+ health with LGBTQ+ lived experience of health and social care provides a more complete approach to setting research priorities.

We identified a lack of involvement of people that identify as LGBTQ+ in healthcare research priority setting. Prior to applications for research funding CLS wanted to orientate research ideas towards topics that were considered important and reflect published accounts of research priority setting in LGBTQ+ healthcare.

## Methods

From a methodological perspective, the growth in reports and descriptions of research priority setting that involve the general public provides more evidence and analysis of different frameworks and approaches, however how these exercises were conducted and who was involved is often under reported making it difficult to assess the exercise and outcomes. Nine common themes of good practice in priority setting [17] and more recent REPRISE guidance [18] for reporting accounts of research priority setting helped the team to agree a short set of criteria for appraising published research priority sets for inclusion in the rapid review.

Our approach to this project was therefore inspired by the James Lind Alliance (JLA) [9] and the Ensuring Value in Health Care Research movement [19], combining inclusive approaches to research priority setting and an evidence informed approach. This translates to a rapid review of published research priority sets in LGBTQ+ healthcare, and the recruitment of relevant public to provide insights and experiences, primarily through an online workshop.

We used the UK Standards for Public Involvement [20] to guide our work with the public, and in reporting this project, we use the GRIPP 2 checklist [21] so that the specific elements of public involvement are described and evaluated.

### Rapid review methods

The search strategy was developed and conducted by a medical librarian (EB). Prior to conducting the searches, the search terms were peer reviewed by another medical librarian according to PRESS criteria [22]. The databases (platforms) Medline (via Ovid), CINHAL (via Ebscohost), Embase (via Ovid), Web of Science (core collection) and PsycINFO (via Ebscohost) were searched from inception to June 2020 with variants of the following search terms, which were in the title and abstract fields, as well as in the subject heading (MESH) term field when these existed in the database. The author team acknowledge the complexity of language associated with gender and sexual identities, that they change over time and that some may be stigmatizing. Our intention with this list of terms was to capture studies that may be relevant to the review acknowledging that some of the search terms reflect historical language rather than current usage.

The Medline search is reproduced below;

((research adj3 (priorit* or agenda* or consult* or consensus)) OR ((patient* or public or stakeholder* or communit*) adj3 (engag* or involv* or empower* or activat* or participat*)) OR (“patient centered research” or “patient centred research”)).ti,ab. OR Health Priorities/.

AND

(homosexual* or gay* or lesbian* or bisexual* or “sexual minorit*” or “sexual orientation” or transsexual* or trans-sexual or transgender* or queer* or “gender fluid” or “gender nonconforming” or LGBT* or non-binary or nonbinary or bisexual or bi-sexual or MSM or MSW or WSW or “men who have sex with men” or “men who have sex with women” or “women who have sex with women” or “gender reassignment” or pansexual or asexual or “sexual identity”).ti,ab. OR exp. Transgender Persons/ or exp. Gender Dysphoria/ or exp. Sexuality/ or exp. Gender Minorities/ or exp. Homosexuality/ or exp. Bisexuality/.

In addition, Google was searched using the following search “research priority setting” AND lgbt (site:.edu OR site:.ac OR site:.gov OR site:.org).

We additionally reviewed references identified from LGBT Magazine Archive and LGBT Thought and Culture Database, searching for the term research priorit*.

We screened abstracts and then full text. Abstracts that only identified health needs rather than research priorities, and sources that identify a very general statement of “more research needed” were excluded. Inclusion criteria for the review were: accounts that identify any LGBTQ+ research priorities; research priority sets based on all methodologies and exercises published in the last 5 years (on the premise that research priorities are time sensitive).

Quality assessment of the priority sets was conducted by (CLS, MR, SC) using adapted from the REPRISE framework. These included; country, population and focus, methods for prioritization, participants in the process, number of priorities, stated strategy/plan for priorities and research-specific calls for action/innovation. We additionally used the Strength of Recommendation Taxonomy (SORT) approach to evaluate the strength of patient-oriented evidence in each paper.

Extracting the priorities and identifying themes was conducted by CLS using an iterative approach, meaning that themes across priorities were developed during the analysis. However, two identified priority sets [23, 24] provided lists of priorities that fell under all of the identified priority themes and for the workshop the team combined these two approaches. (CLS, MR and SC) matching the individual priorities from these two sets independently to the identified themes and following discussion a final amalgamated set of themes with example priorities were agreed for discussion in the workshop.

### Workshop methods

Criteria for people to apply to participate in the process were that they needed to be 18 years and over, identify as LGBTQ+ and either have good health, or use health and social care services. Experience of chronic and long-term conditions, disability and caring roles were also highlighted. Potential participants needed to be able to speak, read, and understand English. We did not attempt to include those who did not speak and understand English. We limited recruitment of researchers (in LGBTQ+ research or other) as we felt that they already have the potential to influence LGBTQ+ research priorities through their work and publications. We were primarily interested in the views of non-researchers that are less likely to have this influence.. Recruitment was UK wide, from urban and rural settings and we prioritised recruitment of participants from black, Asian and other ethnic minority backgrounds.

Recruitment took place over 3 months, following ethical approval for the work from the University of Cambridge Psychology Research Ethics Committee (Application No: PRE.2020.112). A ‘stakeholder map’ was developed of local (to Cambridge) and national LGBTQ+ community groups and organisations. This was followed up with formal (use of mailing list) and informal (conversations and social media) networking by (MR, SC) and an advert was placed in the ‘People in Research’ matching website [25].

We specified that ideally participants would have access to a device (laptop, tablet) to join the workshop (as a mobile phone experience would not be optimal), however this was not a barrier to participating. Participants were offered £100 for 1 h of preparation, taking part in a 2-h online discussion and 1 h follow up activity, this is in line with guidance from NIHR Centre for Engagement [26].

Project information was sent to interested people and organisations with a deadline to respond. Interested candidates were encouraged to make personal contact with the organiser (SC) helping to establish relationships, identify needs to participate fully and troubleshoot/rehearse technical requirements.

Selected participants were sent via email pre workshop materials including; participant information and consent, a workshop programme, an exercise in personal ranking of research themes identified during the rapid review (Additional file 1), a participant list (including people’s interests and motivations to take part), and a ‘wellbeing document’ acknowledging that for some participants the discussion might prove painful or triggering and how to address that (Additional file 2). Material was provided in alternative formats to support inclusion of participants with specific disabilities.

A slide set for use during the workshop, and a detailed set of team notes for each step of the process were developed following team conversations. The use of pronouns (e.g., he/him, she/her, they/them) during the workshop was encouraged, but it was not a requirement. Similarly, participants were encouraged to keep their cameras on during the workshop, but again this was not compulsory.

Before the workshop, participants personally ranked the research themes (1 = most important, 7 = least important), considered the research questions in each theme and chose two that they most resonated with. They were encouraged to record their reasons for these choices.

At the workshop, following an introductions exercise and ‘scene setting’ participants split into two smaller groups, with a facilitator, observer/note taker in each. Another member of the team provided technical support across the two groups and was available for any other issues during the workshop. Facilitators had prepared to maintain a safe space for discussion and ensured that all participants had their say on what was important to them.

Participants referred to their workshop preparation and discussed their top two themes. Facilitators made a note of important themes allocating pre-agreed scores (Number 1 theme = 2 points; Number 2 theme = 1 point). Facilitators encouraged participants to share issues that were considered important, but missing from the research themes list. Facilitators ranked the themes accordingly and shared their screens, inviting comments. Ranked scores were combined across the two small groups and the resultant priority themes were assembled during a short break in the workshop. The whole group appraised, discussed and challenged the overall results. Scores were not changed at this stage, but challenges were heard and discussed. The process concluded with sharing next steps, assessing interest in these and thanks to participants.

Following the workshop, the results were analysed by the research team using a narrative summary approach and were shared with participants, who were invited to comment and add to the themes. Some also sent in their pre workshop notes on the themes providing further context and rationale for their choices.

## Results

### Rapid review results

**Fig. 1:**
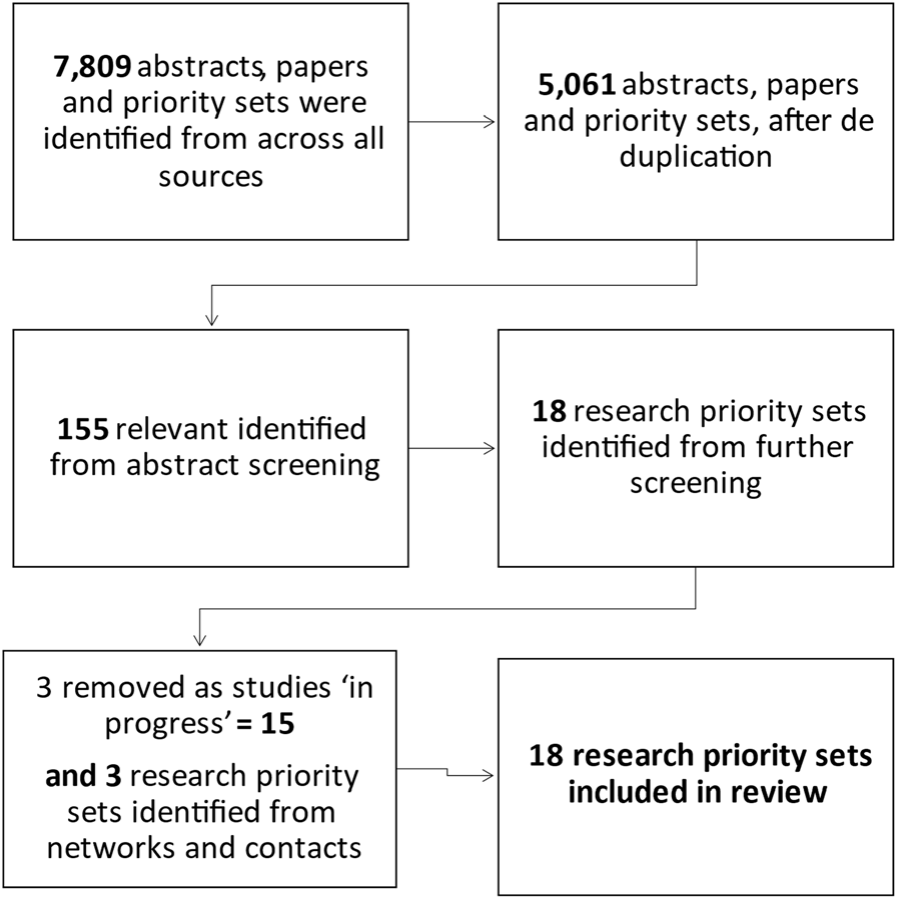
Flow chart of literature search and results

Initially 7809 abstracts, papers and priority sets were identified from across all sources, following de duplication this reduced to 5061. Of these 155 were relevant identified from abstract screening. Further screening of full texts reduced this to 18 relevant reported research priority exercises of which three were removed as they were ongoing studies [27-29]. This resulted in 15 relevant studies for the review. After the workshop while writing up this report three additional relevant priority sets were identified and added in, resulting in 18 relevant studies. The three reports added later were not appraised by workshop participants and did not form part of the framework used in the workshop, however we include them for completeness (Fig. 1).

Of the 18 research priority sets included, four had a broad sexual and gender minority health focus [30-33] three concerned young people (plus school contexts) [34-36], three focussed on older people [37-39], one on lesbians [40] and one on family units [41]. Six specifically addressed transgender and gender non-conforming populations [42-47] (Table 1, included at the end of this manuscript).

**Table 1:** Data extraction and quality assessment of included Research Priority Sets

Paper in date order	Population / focus of research	Methods used	Participants in the process?	Priorities and/or research questions	Stated a strategy, action plans for priorities?	Prioritisation focus included research methodology action or innovation
**Perales, Reeves et al. 2020** Australia	Family lives – of Lesbian, Gay and Bisexual People	Literature review	Co-authors only	10	No	Yes
**McDaid, Flowers et al. 2020** Canada/UK	Gay, bisexual and other MSM/Sexual health literacy.	Consensus building workshop and secondary analysis of two focus group studies	Researchers, service providers, policy makers & knowledge users (38) in HIV prevention, sexual health and health literacy.	15	No	Yes
**Follmer, Sabat et al. 2020** USA	Disclosure of stigmatising identities in the workplace	Literature review Articles written in English	Co-authors only	4	No	Yes
**Clark, Capriotti et al. 2020** USA	Mental health professionals / supporting sexual and gender minority health	Web survey	Mental health professionals (163)	7	No	Yes
**LGBT Health 2020** USA	Sexual and Gender Minority Youth	None stated	Journal editors	13	Yes	No
**LGBT Health 2020** USA	Sexual and Gender Minority Health Research	None Stated	Journal editors	14	Yes	No
**Westwood 2019** UK	Older LGBT people	Scoping study / literature review and workshop	Co-authors only	5	No	Yes
**Westwood 2019** UK	Older LGBT people/abuse	Interviews for commentary and research agenda development	Women (36), Men (24) agedbetween 52–92. Narratives from 17 participants who mentioned abuse.	5	No	Yes
**Poteat 2019** International	Trans communities	Research presentation	Unknown	3	No	Yes
**Newcomb, LaSala et al. 2019** USA	LGBTQ youth health and family influence	Public symposium, expert consultation and working and writing groups	People from academia, federal health agencies, youth serving organizations, advocacy organizations, foundations, (family experience) and youth (40)	27 in 6 themes	No	Yes
**Johns, Poteat et al. 2019** USA	LGBTQ youth health and influence of schools	Public symposium, expert consultation and working groups	People from academia, federal health agencies, youth serving organizations, advocacy organizations, foundations (school experience) and youth (40)	36 in 9 themes	No	Yes
**Diamond 2017** USA	Lesbians/ Relationships	Personal commentary	Author	3	No	Yes
**Olson-Kennedy, Cohen-Kettenis et al. 2016** USA	Gender nonconforming and transgender youth	Review and report of working group (TransNet)	Endocrinologists and other medical professionals experienced in the care of transgender individuals.	10	No	No
**Safer, Coleman, Hembree 2016** USA	Transgender health and medicine	Account of creation of TransNet group	Endocrine Society research priority group, paediatric endocrinology expert in transgender medical researchers.	5	Yes	No
**Feldman, Brown et al. 2016** USA	Transgender/ Medical outcomes research	Review of research to date	Co-authors (reviewing 68 published research papers)	18 research questions in 5 themes 20 priorities in 3 themes	No	Yes
**Papers retrieved and assessed after the workshop**						
**Marshall et al 2019** Canada	Transgender, Non-Binary and Gender Diverse	Literature review and evidence map All types of research explored.	Co Authors	37 Study Topics with a ‘Top Ten’ and ‘Bottom Ten’	No	Yes
**Nagington, Dickinson et al. 2017** UK	Older LGBT people	Symposium, survey and agreement analysis	Survey; 258 participants Symposium; 73 people: 41 from academic institutions (inc. 7 students), 23 from third sector organisationsfrom health and social care, 4 ‘other’	60 research questions in different priority order groups	No	No
**MacCarthy et al 2015** USA	Adult transgender health	Literature search of quantitative studies and gap analysis 1981–2013	Co-authors reviewing 647 published and peer reviewed abstracts in English	6 research gaps	No	Yes

All the included research priority sets originate from English speaking, high and middle- income countries (UK, US, Canada, and Australia), and date from 2016 onwards. Their prioritization approaches ranged from personal commentary, literature reviews, expert workshops, surveys, to ‘in person’ public consultation and meetings and workshops. Participants involved in setting priorities mostly included research academics, health practitioners and advocacy organisations. We noted that two studies involved LGBTQ+ public in their process and four provided consistent patient-oriented evidence (SORT criteria A).

Some exercises focussed on small numbers of priorities [3], others had much larger sets requiring themes and further categorisation.

Of note is that only three exercises had an explicit strategy, action or implementation plan for their priorities, these were direct commissioning (papers for research articles, systematic reviews and meta-analyses) calls, and a special journal issue.

The majority of the exercises [13] suggested methodological developments such as; new and more sensitive theoretical frameworks, increased implementation science studies, recruiting more minority participants in studies, larger and more diverse research cohorts, and research in outcome measures. Several called for more inclusion of LGBTQ+ people in research priority setting and other aspects of research design.

The included priority sets yielded 123 identifiable priorities for research. Numbers of priorities and their specificity varied across reported priority sets. Some were one word e.g., ‘*depression’*, others were more specific ‘*identifying points of intervention in the pathways through which minority stress impacts depression, anxiety and suicidal ideation and behaviours’*. Some of the shorter word priorities had descriptive context and considerations for researchers and research funders.

There were common themes across the priorities including: research methodology and theory development (e.g. prevalence, comparative designs); intersectionality (e.g. race/ethnicity, deprivation, age, trans health priorities (e.g. healthcare access and quality); health literacy; LGBTQ+ health in relation to families and other settings (e.g. school); health care delivery; health promotion and ill health prevention; mental health, emotional wellbeing and stressors; relationships**;** policy evaluation; and sexual health and relationships. The group decided to put to one side priorities concerned with research methodology and theories as this wasn’t in the remit of the study.

The final themes agreed from the research priority sets identified pre-workshop (CLS, SC and MR) were: Health services delivery; Health and social policy; Preventing ill health; Developing or evaluating treatments and interventions; LGBTQ+ specific issues; Particular challenges/intersectionality and Health condition specific.

### Workshop results

#### Workshop recruitment

Initially the aim for was 10 people to take part, however the recruitment process resulted in 17 potential participants so we expanded our planning to accommodate everyone that applied. On the day 14 took part as three sent apologies beforehand (one for personal circumstances and 2 were work related). Characteristics of participants were diverse and are described in Table 2;

**Table 2:** Characteristics of 14 workshop participants

Gender	Six identified as female, one as non-binary and seven identified as male.
Ethnicity	Participants self-identified as Black, Asian or Minority Ethnic (4), British Bangladeshi (1), Latin British (1) Asian British (1) Mixed Race - White & Asian (1)
Age range	21 years – mid sixties
Disability	Two participants declared their registered disabilities, others described living with life-long or ‘chronic conditions’.
Geography	Six participants were from Cambridge and eight from areas in the UK outside of Cambridge.
Interests and motivations	As part of the recruitment process people were asked to state areas of interest in LGBTQ+ healthcare. Topics included; accessing health services, inclusive care homes, end of life care, young people’s health, parenting, mental health, discrimination and bullying.
Other	Three participants were researchers or research students. Several had previous experience of being involved in national and community healthcare research.

There was an intention to have a diverse project and workshop team and this was achieved.

Of the 14 that took part in the project, eight remain connected to the team, and interested in future developments, some have participated in the preparation of this manuscript.

#### Workshop consensus

The seven themes were ordered into priorities by workshop participants. Firstly, in two smaller groups then a combined list was appraised. The small group results were similar with the identical 1st and 3rd and 7th themes, but also some differences. The collective top three research themes were ‘Health Services Delivery’, ‘Prevention’ with ‘Particular Challenges /Intersectionality’. Table 3 (included at the end of this manuscript) contains the full rank order the research themes with additional comments from workshop participants.

**Table 3:** Prioritized research themes

Themes in priority order Points indicate aggregated ranking scores across two small groups	Key reflections and different topics within each theme
**Health services delivery** 16 points	**Primary care emerged as a very important part of health services delivery, and often the ‘first port of call’, therefore sensitive and helpful interactions were considered important areas for research improving experiences.** • Primary care (GP) is the front door, coming out can be exhausting and potentially risky • Training, guidance and support for front line workers (primary care included) in health and social care • Balance between making sure you acknowledge LGBTQ+ identity (and don’t project heteronormative assumptions), also don’t focus entirely on LGBTQ+ identity in service provision. • Mental health support needed, for example domestic violence support lines • Focus on older people is missing in this theme and add in care homes as a feature of services, stories about LGBTQ people in care homes being marginalized • Social care and fostering and adoption overlooked
**Preventing ill health** 11 points	**Preventing ill health is a broad topic, including areas of cancer screening to violence, substance misuse and stress. Intersectional issues were thought to be generally under reported and addressed.** • Under reporting of violence especially in transgender community • Substance and alcohol misuse is a big issue for LGBTQ+ people • Stress from being part of a minority group – leading to mental health crisis • Cancer screening • Reducing HIV
**Particular challenges /intersectionality** 8 points	**Race, ethnicity and the impact of socioeconomic status were the most commonly cited and discussed challenges in intersectionality.** • Mixed race, social class (working class often not represented in LGBTQ+ work) • Lack of emphasis on BAME communities being LGBTQ – stigma in particularly Asian communities about coming out • Neuro diversity and disability • Double stress of racism and homophobia/transphobia • Disparities between black and white trans people are significant • So many inequalities added onto being homosexual including race, ethnicity • Experiencing racism at work, in treatment, homosexuality on top of that feels like you are • Queer trans and intersex people of colour need to be part of the research conversation, otherwise intersectionality will not be taken seriously • Socioeconomic inequality has led to multigenerational health issues • LGBTQ elderly care could be included in this category as age intersects with sexuality? • These issues have a massive influence in affecting how people experience healthcare
**LGBT+ Specific issues** 5 points	**Focus on youth in this theme with the prevention of future health issues with early and appropriate interventions and policy that addresses homophobia and bullying (e.g., in school and care settings).** • Sense of isolation overlaps with mental health issues which continue to be very high in LGBTQ+ young people. • Body image and pressures to conform to LGBTQ+ stereotypes • Eating disorders, under discussed but very important topic that has lifelong implications • Intervening in younger years such as preventing suicide etc. but timing is crucial and flexible methods are essential.
**Developing or evaluating treatments & interventions** 1 point	• Tensions between family and schools an assumption that they all work together – they don’t! • Person-centered treatments important – should be targeted to how we live our lives.
**Health and social policy** 1 point	**Whilst some participants felt distant from policy and its implications for LGBTQ+ people, there were strong views in connection to racism and policy, inadequate counting of LGBTQ+ people and policy in settings such as prisons.** • Policy can shape lives so important to understand what helps and what doesn’t – however Health and Social policy is complicated which drives which, the undercurrents or the policy? E.g., the equal pay act was enacted years ago, but women still don’t have equal pay. • You need something in addition to the policy, such as the general demand in society to make things better. • Focus on mental health • Poor ‘counting’ which means difficult to assess and prevent ill health in this group (see similar comment in LGBT+ specific issues) • Settings such as prisons, immigration centres important • Stronger focus on how structural racism and colonialism impacts LGBTQ+ health care and health care options
**Health condition specific** 0 points	• Are there disparities in provisions for older LGBTQ+ people with health conditions? • Alzheimer’s research going on at the moment, will be interesting to see how it intersects with queer identities • Mental health

For priority theme 1, ‘Health Services Delivery’ there were common experiences of challenges *“Primary care (GP) is the front door, coming out can be exhausting and potentially risky”*. Research into effective training, guidance and support for front line workers in health *and* social care was considered important. The balance of ones’ identity being acknowledged, but not pathologized was also clear, *‘being LGBTQ+ is not an illness’*. Mental health was singled out as very important especially in relation to domestic violence. Finally, a focus on older people (and inclusive care homes) was felt to be missing in this theme.

For ‘Prevention’ research, (priority number 2), many participants wanted to improve the overall health of LGBTQ+ people, for example with cancer screening and HIV prevention. Of particular note was participants’ reflections on issues of bullying and violence, substance misuse and stress which were thought to be generally under reported and addressed. As one participant said *“We are here, we have feelings, I need to be cared for sometimes, I want to be able to breathe” “it feels like ‘living in a world of isolation”.*

The particular challenges and intersectionality (priority 3) in LGBTQ+ healthcare experiences were vividly described in the workshop, with particular emphasis on race and inequalities. Additionally, stigma particularly in Asian communities, living with neuro diversity and disability were cited as areas research could usefully address. Experiencing racism at work, or in accessing healthcare with *“homosexuality on top of that feels like you are the lowest of the low’.* Finally, people of colour needed to be part of the research conversation, otherwise intersectionality between gender and sexual identity and race would not be taken seriously. “*We need a stronger focus on how structural racism and colonialism impacts our health care and health care options”.*

Other commentary on the themes ranged from inclusion of more working-class LGBTQ+ public in healthcare research, recognising the role of “*factors with roots outside healthcare settings (such as criminal justice, care homes, reporting of violence etc.) for an effective investigation”.* There were also observations about different types of research such as population studies where *“Poor ‘counting’ which means it is difficult to assess and prevent ill health in this group*”.

Despite achieving general agreement or consensus of the top research themes across the two small groups some people’s priorities weren’t reflected in the top 3. The workshop framework accounted for this and individual challenges to the priority order were discussed, once the whole group reassembled. One example was the lower rank of the research theme ‘Health and Social Policy’. One participant who was familiar with this area through activism was disappointed that others hadn’t seen this as an important research theme. An engaging discussion about the proximity of policy to people’s lives took place and the challenger was content that their point of view had been discussed. There was a similar debate about the different research issues associated with gender non-conforming and transgender needs as distinct from those of lesbians, gay and bisexual people. This is reflected to some extent in the findings in the literature review where transgender research priorities were considered separately in some studies.

The facility for individuals to challenge the results of a group priority setting exercise is an important methodological feature. Whilst these discussions were facilitated, the rank order of research themes weren’t open for re-ranking. Authors were encouraged by the candid and frank exchange of views in the group, who were never intolerant or disrespectful of each other. The workshop closed with participants content with the outcome.

#### Workshop experience

Participants reflections on their experience of being part of the project and the workshop were submitted during the workshop verbally or in the chat function and after via email.

*“It was a great workshop, thank you so much. I must say, it was one of the best conducted ones I have attended, from start to finish”.* Clara, workshop participant.

*“I'd like to be forward in thanking you for hosting a culturally diverse group. Whether this was organic or thought about doesn't matter; it was extremely welcomed and was very different from other LGBTQ projects/spaces I've been involved with in Cambridge.”* Jade, Workshop participant.

*“I have been involved in Patient and Public Involvement and Engagement (PPIE) for over three years and this is the first invitation I have had to explore LGBTQ+ matters”.* Rumi, Workshop participant.

Researcher and facilitator reflections are also important in exercises such as these, including reflection on how much does identity reflect our approach to the exercise and how we interpret the results?

*“as a straight woman facilitating the process, my role is to support and enable participants to speak their truth. I have no vested interest in the outcomes but care greatly about how they are achieved”*

*“I learned more about is the critical importance of sensitivity and recognition of the tensions people might (very legitimately) bring from difficult experiences/bias/discrimination. I really noticed that use of language, pre-briefings, use of pronouns, pre briefings and careful preparation by the team etc all had an impact on helping participants feel safe and respected and therefore able to express views/discuss/disagree without any difficulties”.*

*“It has felt extremely validating to work on a project that genuinely puts LGBTQ+ voices at the centre of the research but doesn't put the burden of labour on LGBTQ+ people. It often feels like the only way for LGBTQ+ research to get done is for the LGBTQ+ community itself to initiate, advocate and keep the momentum going, so it was lovely to be part of a project that fully engaged with the community and amplified the LGBTQ+ experience without demanding extensive work from the participants. I felt like a really valued member of the team, and felt my perspective as a queer person was being taken into account at every stage. The thoughts and experiences shared in the workshop and the paper feel very true to my own experience of healthcare and I'm pleased that we've been able to capture them in a tangible and useful way.”*

## Discussion

We carried out a rapid review of published accounts of research priority setting in LGBTQ+ healthcare. The results of this informed an online workshop with 14 members of the public that identified as LGBTQ+. In this workshop a consensus was agreed on a priority order of 7 research themes and their associated questions. Priority themes were Health Services Delivery, Prevention, and Particular Challenges and Intersectionality; the other four were prioritised less highly but were nonetheless still identified as important within the exercise. We recognise that this was a relatively small group of people, and that their views will be shaped by their personal experiences, the current sociocultural climate, and maybe even the COVID 19 pandemic. Within each theme there was variety of specific questions and points raised by workshop participants. Workshop participants highlighted the inter connectedness of themes, for example, there were overlaps in themes especially between Intersectionality [3] and LGBTQ+ Specific [4] themes where mental health featured in both.

A strength of this exercise has been assembling a set of published existing priority sets in LGBTQ+ research, which shows the breadth of approaches used to establish priorities and more understanding of the nature of participants in these processes, and the breadth of areas where research is had been identified as required. Having developed the themes independently through the rapid literature review we noted that they have a lot of overlap with the research areas defined by the Health Research Classification System which encompass all aspects of health-related research activity [48].

It has also pointed to a ‘western’ view of LGBTQ+ research, with all the priority setting processes focussing on high- and middle-income countries. This fits with the Western, Educated, Industrialized, Rich and Democratic (WEIRD) [49] bias in social science research. It is worth noting that although included priority sets were from 2016 onwards, earlier priority sets relating to research priorities in HIV/AIDS from low- and middle-income countries might have addressed this bias.

Another strength is the ethnic diversity, age and inclusion of people that experience disability in different forms that took part in the workshop. We set out to recruit people from different backgrounds, ethnicity and contexts, whilst acknowledging the WEIRD context of the prioritisation process, and this was achieved. The rapid literature review showed a tendency not to involve LGBTQ+ non-researcher public in healthcare research priority setting; many participants mentioned the novelty of their experience in this regard *“LGBTQ+ folk are overlooked and this definitely could be pursued”.*

Strengths in the process were noted by several contributors, such as the critical importance of sensitivity and recognition of the tensions people might bring from difficult experiences, discrimination and homophobia. The careful use of language, pronouns, expectation management and preparation by the team *“all had an impact on helping participants feel safe and respected*”. This helped people to express their views, discuss and disagree without any difficulties. The financial incentive to take part helped people commit to the process in terms of their time and they were generous with extra feedback and insights subsequent to the workshop.

There were weaknesses in this exercise**.** We started with the results of a rapid literature review, rather than from where participants wanted to start [50]. Many priority setting exercises gather ideas for research themes initially and then prioritise these. This doesn’t discredit our approach and asking for comments on the themes and ‘missed issues’ helped balance up our quicker method. From a methodological perspective, the themes were presented in the same order to all workshop participants; it is possible that this had an unconscious influence on the prioritisation outcomes, although hopefully mitigated through the wider discussion part of the workshop.

We did not involve our participants in the rapid review process due to the scheduling of the project, however some members of the project team identify as LGBTQ+ and provided expertise in relation to search terms, journals etc. and critique on the results. CLS and SC also corresponded with international authors in LGBTQ+ research to inform aspects of the project. Recent research databases such as ‘knowsy’ [51] will further support work in this area.

We did not attempt to include those who did not speak and understand English, due to the small scale of the project and funding. We stated in recruitment materials and processes that applicants needed to ‘identify under the umbrella of LGBTQ+’. On contact we did not ask them to disclose their identity, but all did. By not seeking participants that have not disclosed their sexual or gender identity is a potential limitation to interpreting our results. The low number of younger people involved in this process is a limitation and might have influenced the nature of priority topic areas identified.

We note that trans healthcare can be very different and separate from LGBTQ+ community health issues generally and that transgender perspectives are represented more fully in the results of the rapid review than from the workshop.

Some participants joined via mobile phone; whilst this wasn’t a barrier to take part it was potentially less satisfying for them. Some participants didn’t come on camera for the workshop, again this wasn’t a barrier to take part but it did make it harder for the facilitators and other participants to connect. Conversely being off camera may have made it easier for some people to take part especially if they weren’t ‘out’ to others about their identity. With a relatively small number of ‘people in the room’ the outputs were always going to reflect the priorities of those taking part. However, as the starting point of the workshop reflected the current published evidence from research priorities in LGBTQ+ research perhaps this represents a balance of approaches and outcomes? Finally, the presentation of the themes (Additional file 1) was on the whole descriptive, but in one instance a theme hinted at the result from the rapid review and this may have biased participants view of the themes.

There are implications for LGBTQ+ research from our exercise, both in terms of identified priorities that future research should focus on and on a practical level making sure that the work from this exercise is used and taken forwards CLS is pursuing these currently as an individual researcher. These prioritised themes are also more widely relevant for research funders and commissioners. The second set of implications from this research are in setting a template and reflecting on considerations for involving LGBTQ+ public in strategic research conversations.

## Conclusions

Health services delivery, prevention and the intersectionality of sexual orientation and gender identity with other disadvantage were highlighted as research priorities for LGBTQ+ health from workshop participants in this shortened research prioritisation exercise. The challenge now to the research team, the wider LGBTQ+ research community and to research funders is to commission, plan and carry out research that addresses these priorities.

## Supplementary Information

**Additional file 1.** Pre workshop ranking form.

**Additional file 2.** ‘Your Wellbeing’ sheet.

**Additional file 3.** GRIPP2 short form.

## Data Availability

No additional data or materials are available.

## Abbreviations

LGBTQ+: Lesbian, Gay, Bisexual, Transgender and Queer, the + denotes other dimensions of sexuality and gender that may exist
MSM: Men who have Sex with Men
