# Supplementary material for: Are we asking the right questions? Working with the LGBTQ+ community to prioritise healthcare research themes
Source: Res Involv Engagem. 2021 Sep 24;7:64. doi: 10.1186/s40900-021-00298-7 (PMC8460395; doi:10.1186/s40900-021-00298-7)
Supplement: Supplementary file 1 — Additional file 1. Pre workshop ranking form. [file 40900_2021_298_MOESM1_ESM.pdf]

Document 2. LGBTQ+ healthcare research – are we asking the right questions?

Individual ranking of 7 research themes for workshop on 29th October 2020

This is a list of 7 themes for healthcare research in LGBTQ+ populations, with some questions in each topic. They have come from a review of published research about LGBTQ+ healthcare research priorities done by Katie Saunders, the researcher who is interested in your opinions and thoughts. There is more information about the themes and the review at the end of this document.

Before the workshop can you **rank the themes from 1 – 7** (1 being the *most important* to you) and for each theme, make a note of the questions that are of most interest to you – there is space to record your thoughts and reasons for your choices.

The most **important thing** is that you **rank the themes** and **read all of the questions,** even if you find it difficult to choose your top 2.

Please have this with you when you join the online workshop. Everyone will discuss their choices in small groups.

**Themes** and questions (in bullet points)	**Rank (1-7)**	**Notes on your choices**
**A. Health services delivery**<br>• Guidance for and best practice by health care professionals for conditions of particular relevance to LGBTQ+ people<br>• Best healthcare provided in the local community (e.g. GP) for LGBTQ+ people<br>• Health care discrimination, access to health care, and health outcomes among LGBTQ+ people<br>• Provision of care that helps transgender and non-binary youth align their body with their gender identity		

**B. Health and social policy**<br>• Impact of LGBTQ+ policies (e.g. health) and services on health and wellbeing<br>• Impact of policies and services on LGBTQ+ youth health<br>• LGBTQ+ people to attain their full health potential and not be disadvantaged because of social circumstances<br>• Considerations in conducting healthcare research with LGBTQ+ youth, (for example ethics and research methods)		
**C. Preventing ill health**<br>• Health benefits of sexual orientation and gender identity disclosure and data collection<br>• Stress from being part a minority group, mental health, and health risk behaviours in LGBTQ+ people<br>• Cancer screening and cancer prevention in LGBTQ+ populations<br>• Strategies to prevent and reduce substance use and misuse in LGBTQ+ youth<br>• Reducing HIV and other sexually transmitted infection risks among LGBTQ+ youth		
**D. Developing or evaluating treatments and interventions**<br>• Impact of family and school-based programmes on health and wellbeing of LGBTQ+ youth		

• Health impacts of treatment that helps transgender and non-binary youth align their body with their gender identity, (including surgery and hormones)<br>• Tailored prevention and treatment approaches for substance abuse in LGBTQ+ people		
**E. LGBTQ+ specific Issues**<br>• Identifying things that help (and the best time to intervene) LGBTQ+ youth experiencing stress from being in a minority - especially where depression, anxiety, and suicidal feelings are evident<br>• Where LGBTQ+ youth are experiencing stress from being in a minority, what are the resilience and vulnerability factors and what are the foundations for these factors?<br>• Reproduction options, decision-making, and outcomes for transgender and non-binary youth and young adults<br>• Eating-related health issues in LGBTQ+ youth and adults<br>• Physical and mental health of children of LGBTQ+ parents		
**F. Particular issues/intersectionality**<br>• How race, ethnicity, gender, and sexual orientation together affect health outcomes for LGBTQ+ people<br>• Effects of violence/trauma on mental and physical health in LGBTQ+ youth		

• Health care needs of LGBTQ+ people in prison and other secure settings<br>• Health care for LGBTQ+ youth living out of home (foster care, juvenile justice system, and homeless)		
**G. Health condition specific**<br>• Physical health and chronic conditions disparities in LGBTQ+ people<br>• Alzheimer's disease and other dementias among older LGBTQ+ adults		

Thankyou!

For preparing for the online workshop on 29th October at 9.30 – 11.30

Please have this document with you when you join the workshop.

More information about themes

A. Health services delivery

Health services delivery research is done to improve the evidence of the quality, accessibility and organisation of health services. This will include research done in the NHS and other healthcare settings and research done with healthcare professionals that deliver healthcare services and people that use these services.

B. Health and social policy

The research that is embedded in thinking about or evaluating the impact of local or national policy or legislative changes on LGBTQ+ health.

C. Preventing ill health

This is research aimed at the primary prevention of disease, conditions or ill health, or promotion of well-being

D. Developing or evaluating treatments or interventions

Discovery and development of therapeutic interventions (e.g. treatments) and the testing and evaluation of therapeutic interventions in clinical or non-clinical settings.

E. LGBTQ+ specific issues

These would be research questions for health topics that are specific to LGBTQ+ women and men, or an area where LGBTQ+ adults and young people experience differentially much greater health challenges.

F. Particular challenges and intersectionality

Many of the research priority sets that we identified highlighted that some groups of LGBTQ+ adults and children experienced particular challenges, for example, people who are homeless, in contact with the criminal justice system, or health inequalities associated with ethnicity, or living in the poorest areas of the country.

G. Health condition specific

Research into individual care needs and management of disease, conditions or ill health

How we arrived at the themes and questions

In developing the themes and questions to be explored during this workshop we took a two-stage approach. First, we reviewed research/academic journal articles and non-academic sources to find previous sets of LGBTQ+ health research priorities. In doing this we identified 115 research priorities published in 18 separate priority sets, some were very specific for example looking at specific settings, or ages, or groups. We then identified 7 topics under which most of the priorities fall.

'LGBT Health' is a US journal focusing on LGBT health research. The journal has recently published two sets of LGBT health research priorities – one for adults, and one for young people. Of the initial 18 sets of research priorities identified we took these two sets of questions from LGBT Health for this workshop, because they were recent, covered a wide range of LGBT health topics, and had at least one or two questions which fell under the 7 themes that we identified from across all 18 priority sets in the first part of this work.
