# Supplementary material for: Are we asking the right questions? Working with the LGBTQ+ community to prioritise healthcare research themes
Source: Res Involv Engagem. 2021 Sep 24;7:64. doi: 10.1186/s40900-021-00298-7 (PMC8460395; doi:10.1186/s40900-021-00298-7)
Supplement: Supplementary file 2 — Additional file 2. ‘Your Wellbeing’ sheet. [file 40900_2021_298_MOESM2_ESM.pdf]

Looking after yourself during, and after the workshop

Talking about, and listening to others talking about LGBTQ+ healthcare and research may prove challenging for some workshop participants.

If at any time during the workshop you want to leave – please can tell us you are leaving via the chat box privately to Lucy, and if you want us to contact you leave a number. This way we know that you have left and we can make contact with you after the workshop if you want to talk about anything.

Alternatively, you may want to access LGBTQ+ specific services and some of these have been listed below;

Support and information

Local to Cambridge

http://encompassnetwork.org.uk/pinkguide

LGBT Foundation

You can call LGBT Foundation's helpline on **0345 3 30 30 30** or email us on helpline@lgbt.foundation

Bespoke support for different groups https://lgbt.foundation/who-were-here-for

Stonewall

Information service; Freephone 0800 0502020 (9.30 – 4.30, Monday – Friday)

https://www.stonewall.org.uk/help-and-advice

Mental health

Samaritans. To talk about anything that is upsetting you, you can contact Samaritans 24 hours a day, 365 days a year. You can call 116 123 (free from any phone), email jo@samaritans.org or visit some branches in person. You can also call the Samaritans Welsh Language Line on 0808 164 0123 (7pm–11pm every day).
