# Supplementary material for: Are we asking the right questions? Working with the LGBTQ+ community to prioritise healthcare research themes
Source: Res Involv Engagem. 2021 Sep 24;7:64. doi: 10.1186/s40900-021-00298-7 (PMC8460395; doi:10.1186/s40900-021-00298-7)
Supplement: Supplementary file 3 — Additional file 3. GRIPP2 short form. [file 40900_2021_298_MOESM3_ESM.pdf]

GRIPP2 short form

Section and topic	Item	Reported on page No
1: Aim	Report the aim of PPI in the study	Page 7
2: Methods	Provide a clear description of the methods used for PPI in the study	Pages 12-14
3: Study results	Outcomes—Report the results of PPI in the study, including both positive and negative outcomes	Pages 18-21
4: Discussion and conclusions	Outcomes—Comment on the extent to which PPI influenced the study overall. Describe positive and negative effects	Pages 21-23
5: Reflections/critical perspective	Comment critically on the study, reflecting on the things that went well and those that did not, so others can learn from this experience	Pages 23-24

PPI=patient and public involvement
